# Landscape Patterns in Rainforest Phylogenetic Signal: Isolated Islands of Refugia or Structured Continental Distributions?

**DOI:** 10.1371/journal.pone.0080685

**Published:** 2013-12-02

**Authors:** Robert M. Kooyman, Maurizio Rossetto, Hervé Sauquet, Shawn W. Laffan

**Affiliations:** 1 Department of Biological Sciences, Macquarie University, Sydney, Australia; 2 National Herbarium of New South Wales (NSW), Royal Botanic Gardens and Domain Trust, Sydney, Australia; 3 Laboratoire Ecologie, Systématique, Evolution, Université Paris-Sud, CNRS UMR 8079, Orsay, France; 4 School of Biological, Earth and Environmental Sciences, University of New South Wales, Kensington, Sydney, Australia; Consiglio Nazionale delle Ricerche (CNR), Italy

## Abstract

**Objectives:**

Identify patterns of change in species distributions, diversity, concentrations of evolutionary history, and assembly of Australian rainforests.

**Methods:**

We used the distribution records of all known rainforest woody species in Australia across their full continental extent. These were analysed using measures of species richness, phylogenetic diversity (PD), phylogenetic endemism (PE) and phylogenetic structure (net relatedness index; NRI). Phylogenetic structure was assessed using both continental and regional species pools. To test the influence of growth-form, freestanding and climbing plants were analysed independently, and in combination.

**Results:**

Species richness decreased along two generally orthogonal continental axes, corresponding with wet to seasonally dry and tropical to temperate habitats. The PE analyses identified four main areas of substantially restricted phylogenetic diversity, including parts of Cape York, Wet Tropics, Border Ranges, and Tasmania. The continental pool NRI results showed evenness (species less related than expected by chance) in groups of grid cells in coastally aligned areas of species rich tropical and sub-tropical rainforest, and in low diversity moist forest areas in the south-east of the Great Dividing Range and in Tasmania. Monsoon and drier vine forests, and moist forests inland from upland refugia showed phylogenetic clustering, reflecting lower diversity and more relatedness. Signals for evenness in Tasmania and clustering in northern monsoon forests weakened in analyses using regional species pools. For climbing plants, values for NRI by grid cell showed strong spatial structuring, with high diversity and PE concentrated in moist tropical and subtropical regions.

**Conclusions/Significance:**

Concentrations of rainforest evolutionary history (phylo-diversity) were patchily distributed within a continuum of species distributions. Contrasting with previous concepts of rainforest community distribution, our findings of continuous distributions and continental connectivity have significant implications for interpreting rainforest evolutionary history and current day ecological processes, and for managing rainforest diversity in changing circumstances.

## Introduction

Descriptions of the origins and survival of rainforest on the driest habitable continent on earth have, like the rainforest, shifted through time. The Australian rainforest narrative has swung between concepts as varied as recent invasion by a northern (Indo-Malesian) flora [1] to evolutionary autochthony and the persistence of ancient Gondwanan communities with Antarctic origins [2]. The history of supporting biogeographic explanations shows similar conceptual shifts and swings between long-distance dispersal across vast oceans, to rafting continents and vicariant evolution driven by isolation and divergence [3].

We know from the fossil record that rainforest vegetation still dominated the Australian continent during the Eocene to Early Oligocene (ca. 30 Ma) [4]. The massive contraction that followed occurred as a consequence of palaeogeographic and palaeoclimatic factors [5] that increased aridity and reduced the extent of the ancestral rainforest. During the last ca. 30 Ma increasing aridity [6] interacted with topographic and edaphic gradients [7], [8] and the increasing incidence of fire to shape the area of available habitat for broad-leafed mesic vegetation [9]. By the Early to Middle Miocene (<20 Ma) Australia's moist forests were already contracting toward their current position along the east coast [4]. The lack of major topographic relief reduced the potential buffering effect of altitude (and moist sub-montane habitats) [10] on the impact of aridity and the distribution of rainforest over much of the continent. The result was a rainforest distribution constrained mostly to the eastern side of the Great Dividing Range in eastern Australia, and a reduction in rainforest species and lineages [9], [11], [12].

The accepted and most widely used narrative to explain the distribution of rainforest is that the factors identified above fragmented the rainforest into an archipelago of different sized ‘islands’ isolated by dry-land barriers with abiotic conditions unsuited to rainforest and occupied by fire-tolerant sclerophyll vegetation. The isolated rainforest fragments then drifted towards their current differentiated assemblage compositions according to local levels of disturbance and local circumstances, with relatively little interaction with the surrounding vegetation matrix [2], [13]. We refer to that as the ‘isolated fragments’ hypothesis.

Despite the dominant trend to rainforest contraction, palaeobotanical evidence suggests some alternation between warm-wet (expansion) and cool-dry (contraction and extinction) cycles [14]. The more recent and severe glacial cycles of the Pleistocene exemplify the global climate fluctuations [15] that led to contraction of rainforest across the Australian continent. Re-expansion of rainforest and rainforest elements into surrounding sclerophyll forests dominated by eucalypt species during the warm-wet interglacials [14], [16], [17] provided further mechanisms for localized assemblage reshuffling and population-size enhancements. It also supports the idea that the surviving Australian rainforests and rainforest species were adapted to these expansion and contraction cycles. Perhaps not surprisingly, some of Australia's dry-adapted flora is derived from ancestral moist forest lineages, demonstrating that adaptation and biome shifts were possible, and did occur [18], [19].

At regional scales, diversity gradients and species pool sizes are strongly influenced by both evolutionary and ecological factors. Previous studies of Australian rainforest trees showed that local assemblages were influenced by the distribution and composition of species pools at various scales and by local environmental history [7], [20], [21]. The influence of environmental and ecological factors, and functional traits (including those related to dispersal), on the assemblage of Australian rainforest plant species at plot, local area, regional, and continental scales has been tested using genetic, phylogenetic, and plot-based ecological and functional trait methods [21]–[25]. In addition, how those factors impacted on evolutionary history and local assemblage processes [21], and how the processes differed across regions at continental scales [26], [27] has been investigated for some species and clades. In several cases, research results have suggested more of a continuum of genetic diversity and distributions across broad geographic extents [23], [28]. We refer to that as the ‘continuous distribution’ hypothesis.

The long and varied history of the Australian rainforest has left its mark on the flora, including the signals of 1) persistence, ancient conservatism, and floristic connection to Gondwanan rainforest [19], 2) immigration to and emigration from tropical Indo-Malesia [13], [29], 3) the mixing of conservative rainforest elements with a rapidly diversifying dry-adapted sclerophyll flora across a significant range of habitats [9], [30], and 4) continuing diversification of some rainforest lineages [24]. Elucidating the outcome of the influence of these sometimes contradictory continental scale factors on patterns of diversity in the extant woody rainforest flora is the focus of this paper.

The woody life forms considered here are free-standing trees and shrubs, and climbers (vines). Little is known about the geographic distribution of phylogenetic structure and patterns of community assembly of climbing compared to free-standing species. For example, comparing the different life forms will provide a measure of the diversity of climbing and free-standing taxa in relation to latitude, and overall rainfall patterns [31], [32]. In addition, we know that many free-standing and climbing plant families, genera, and species are shared with Indo-Malesia (inclusive of Papua New Guinea) [1], [29]. However, the idea of low latitude origins (for the Australian rainforest flora) is not well supported by global palaeo-history or current day distributions, despite palynofloras overwhelmingly supporting tropical origins for the angiosperms as a whole [33].

In relation to the isolated fragments versus continuous distribution hypotheses for continental rainforest species distributions we expect: 1) weak phylogenetic structure (neutral values) and low endemism where continental connectivity is uniformly high; 2) strong clustering and low endemism where environmental filtering is uniformly strong; 3) high endemism where there are high levels of speciation in taxa filtered into habitats; 4) strong evenness (over-dispersion) and high endemism where rainforest diversity is restricted to larger refugia; and 5) strong evenness and low endemism where rainforest species from disparate lineages have either expanded their distributions or persisted in localised co-occurring populations.

We tested for those patterns by including whole of continent rainforest species distributions, and defined local assemblage scale by using grid cells rather than assemblage level plot-samples. This provided us with an unprecedented opportunity to improve our understanding of broad-scale (continental) processes. However, we acknowledge that larger scale sampling is less likely to detect the signal of factors such as competition between individuals and co-occurring species. In that regard it is important to highlight that until now the phylogenetic structure of woody rainforest vegetation across continental extents and across all species distributions has not previously been tested.

## Methods

### Background

The Australian rainforest is rich in families and genera and includes basal lineages of many plant clades [13]. There are 668 genera among its 136 woody free-standing (tree and shrub) and climbing (vine and liana) families of seed plants (Table 1; Table S1 and S2 in Appendix S1).

**Table 1:** Number of woody rainforest families, genera and species by region in continental Australia including in-shore islands.

	Trees			Climbers			All woody combined			
Location	Fam.	Gen.	Spp.	Fam.	Gen.	Spp.	Fam.	Gen.	Spp.	IMPNG%
Australian rainforest	111	524	1872	61	166	434	136	668	2306	27
IMPNG (shared taxa)	79	256	448	47	110	182	101	353	630	-
Australian Rainforest Regions										
WA_NT	62	195	354	33	75	123	79	265	477	60
CY	86	317	715	45	125	231	104	426	946	56
WT	102	435	1279	52	139	307	125	557	1586	32
CQ	78	271	626	39	90	165	96	353	791	43
BR	74	260	585	40	81	155	97	336	740	24
SNSW	50	108	158	29	41	60	69	149	218	23
Vic*	34	49	64	23	27	34	52	76	98	16
Tas	19	34	44	6	6	6	25	40	50	0

Trees - free-standing trees and shrubs, and Climbers – vines. Wet and dry sclerophyll (non-rainforest) species, mangroves, herbs, palms (including climbing palms), ferns and cordylines were excluded from this synthesis. IMPNG% - percentage of species shared between Australia (Aust) and Indo-Malesia (IM) including Papua New Guinea (PNG) based on currently available taxonomy. Note that the percentages are based on current taxonomic allocations and do not indicate the origins of the species as being in either IMPNG or Australia. Regions include (from lower to higher latitude in an arc from northwest to southeast): WA_NT - West Australian Kimberley Region and Northern Territory; CY - Cape York; WT - Wet Tropics; CQ - north to central Queensland (including Eungella Range and Proserpine area); BR - south-east Queensland and northern New South Wales (Bundaberg to the Hunter Region); SNSW - southern New South Wales; Vic* – Victoria; and Tas - Tasmania. * includes part of SNSW.

### Species distribution data

**Figure 1:**
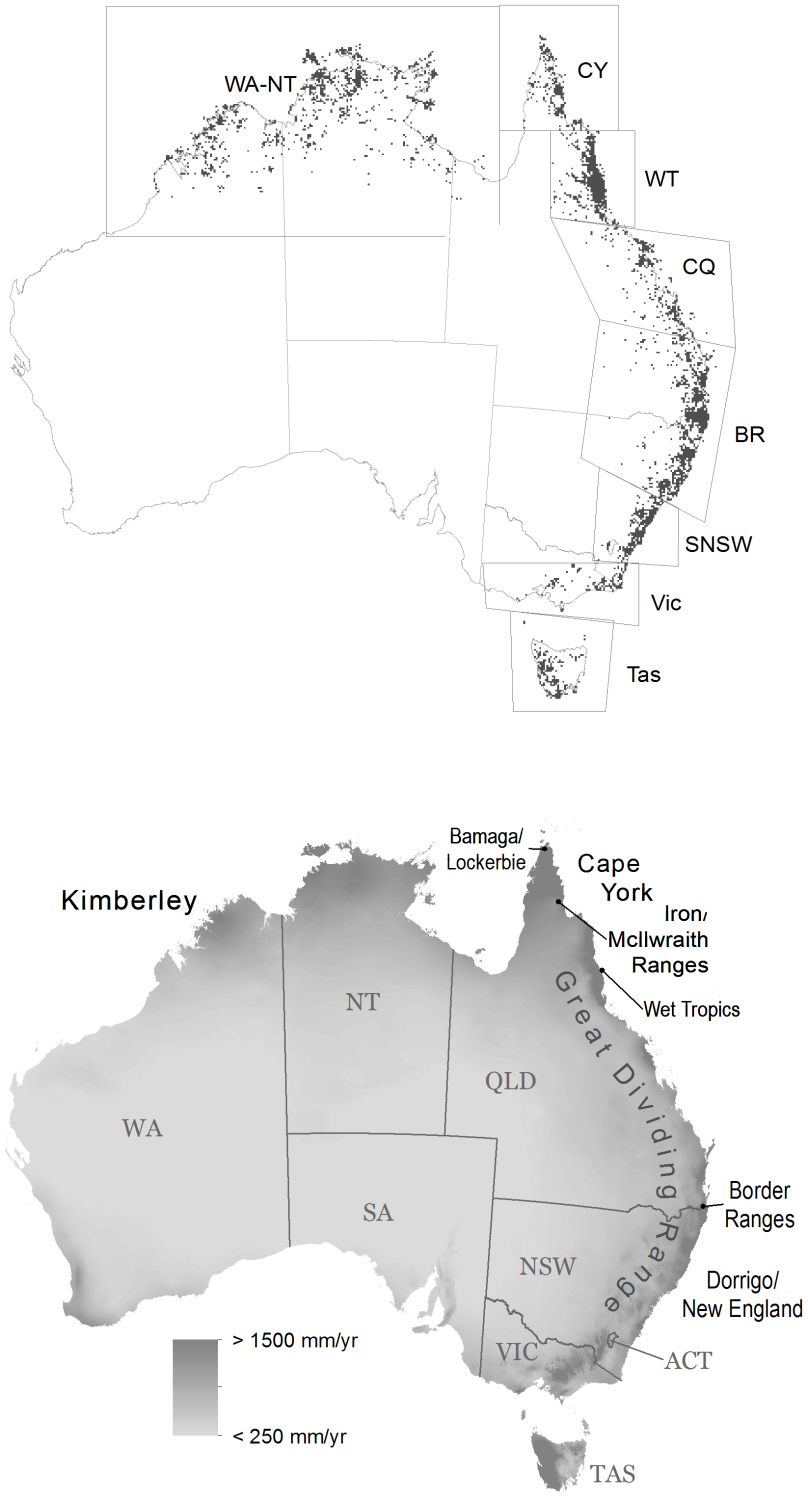
Australian rainforest woody species distribution, and continental rainfall. Top: Rainforest species distributions by region shown here as range 6 - 464 species (see PD in Fig. 2) in 10×10 km grid cells in Australia based on Australian Virtual Herbarium records. Bottom: rainfall map of Australia showing details of coastline and near shore islands with place names and states. Regions from northwest to southeast are WA-NT - Western Australia and Northern Territory; CY - Cape York; WT - Wet Tropics (WT); CQ - central Queensland; BR - southeastern Queensland and northern New South Wales; SNSW - southern New South Wales; Vic – Victoria; and Tas - Tasmania. Additional state name abbreviations not explained in region names are Qld - Queensland; SA - South Australia.

**Figure 2:**
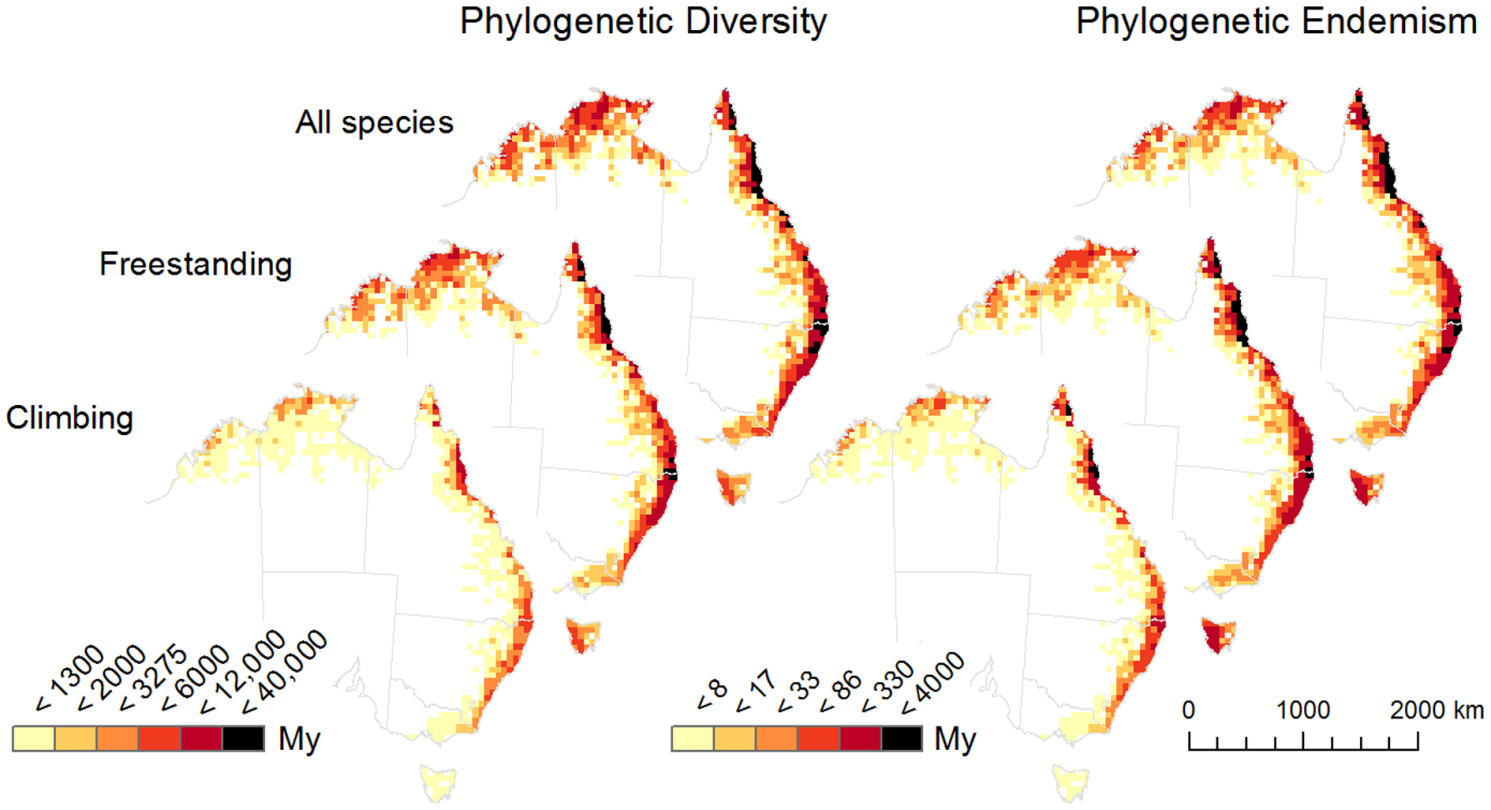
Phylogenetic diversity patterns. Phylogenetic Diversity (PD) and Phylogenetic Endemism (PE) values for 50×50 km grid cells for all woody plants combined, and for two growth forms representing freestanding (trees and shrubs), and climbing (vines) plants. For clarity of presentation cells are clipped to the coastline and off-shore islands are not displayed. Darker colours (red to black) and higher values reflect greater concentrations of evolutionary diversity, particularly for PE. PD is highly correlated with species richness so can also be read as a ‘heat map’ for richness.

**Figure 3:**
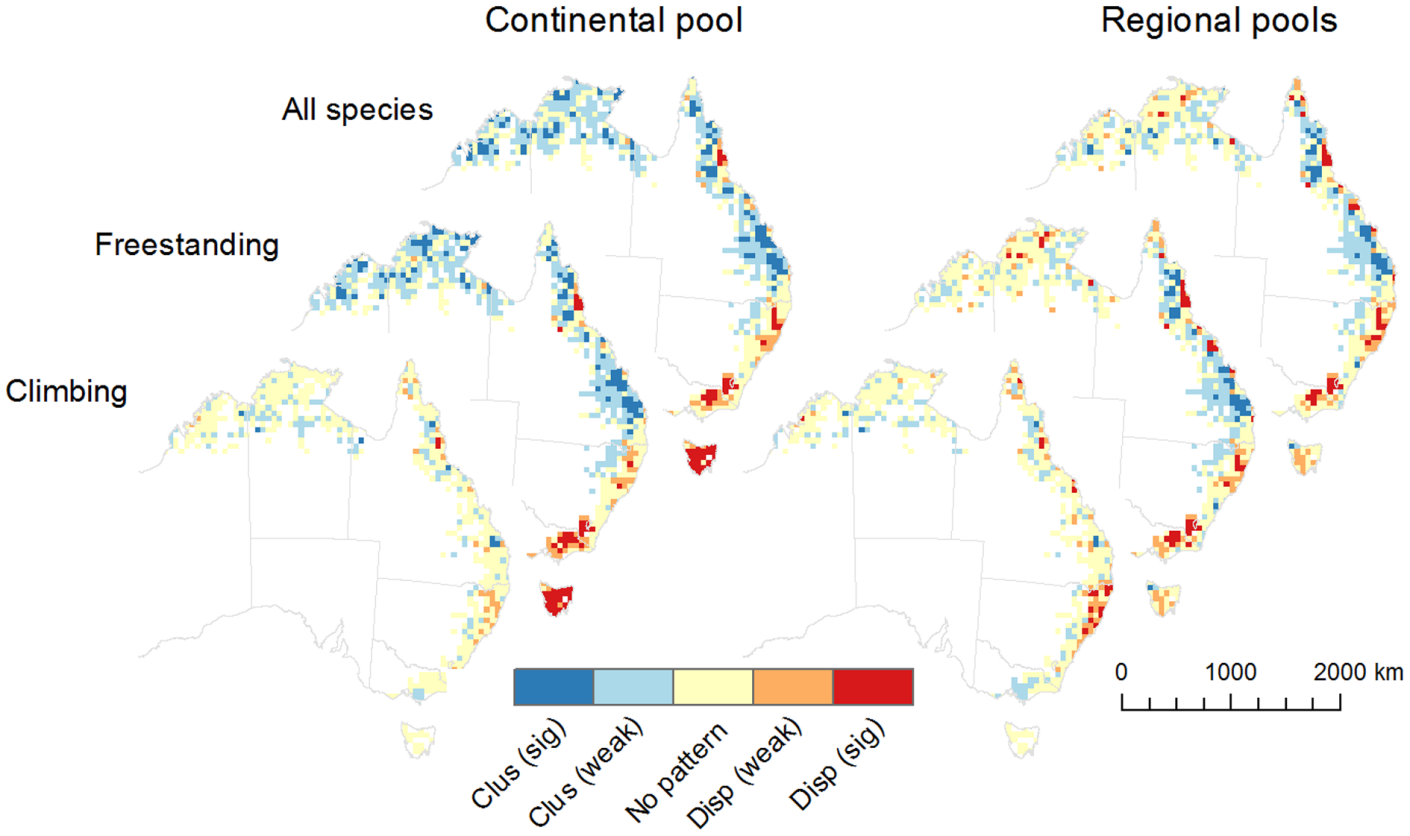
Phylogenetic structure of Australian rainforests. Net Relatedness Index by grid cell (50×50 km) based on continental and regional species pools for all woody plants combined, for freestanding (trees and shrubs), and climbing (vines) plants. For clarity of presentation cells are clipped to the coastline and off-shore islands are not shown. Clus (sig) - significant clustering; Clus (weak) - weak clustering; Disp (sig) - significant evenness (over-dispersion); Disp (weak) - weak evenness (over-dispersion); No pattern - no phylogenetic structure (relative to null model).

We used a taxonomically and geographically comprehensive data set representing ca. 180,000 georeferenced records for 2306 rainforest species, including seasonal monsoon and drier vine forest species [34]. The 1872 free-standing and 434 climbing plant species in the data set represent all of Australia's woody rainforest taxa. Marginal rainforest species (i.e., species that occur mostly on the edges of rainforest in moist and dry sclerophyll and mangrove communities) were excluded from the synthesis presented here. Species selection was comprehensive and provides a complete representation of woody rainforest diversity, but was very conservative in relation to including taxa from other communities. This data set is focused on rainforest species distributions and is not contingent on an *a priori* definition of rainforest and rainforest community distribution. For analysis, the georeferenced species records were first aggregated to presences in 10×10 km grid cells using the Biodiverse software [35] (Fig. 1). These grid cells represent ‘assemblage’ level data for rainforest species across the continent. The 10×10 km grid cell assemblages were aggregated into 50×50 km cells once it was determined that patterns remained the same across these scales. Some dry vine forest species occur as isolated trees in very low rainfall (non-rainforest) areas. To avoid populating drier (non-rainforest) inland areas with cells and potentially over representing the spatial distribution of rainforest in figures (Figs 1–3), only grid cells with >5 species were included. Higher minimum thresholds resulted in reduced representation of species-poor rainforest in southern regions such as Tasmania and Victoria.

### Taxonomic compilations and phylogenetic supertree

Taxonomic checking used currently available and updated online data base resources [36]. All data sets were reconciled with collection records using the Australian Virtual Herbarium (AVH) database [37], the Angiosperm Phylogeny Group (APG III) classification [38], the Taxonomic Nomenclature Checker – GRIN [39], and The Plant List [40].

A phylogenetic supertree was generated using PROTEUS [41]. The procedure, similar to Phylomatic [42], combines the phylogenetic backbone of seed plants as summarized on the Angiosperm Phylogeny Website [43] with internal phylogenetic structure of families where available and assuming that genera are monophyletic. Although this assumption might prove incorrect for some genera in the future, it is a reasonable and necessary approximation for the purpose of this study. In addition, we were careful to follow the latest family-level phylogenetic classifications and genus concepts in PROTEUS in order to limit the extent of this problem to the best of our knowledge. Where undescribed species within genera were included they were allocated a collection location name so trait values and distribution records could be allocated to them. Conspecific taxa within the original data compilation were all resolved to a single name. Varietal status was merged for combined trait and phylogenetic analyses (not presented) as it was acknowledged these would be absorbed into polytomies. The tree is available in PDF form as Appendix S2.

In order to include branch lengths in our analyses of phylogenetic endemism, diversity, and structure, we then transformed this supertree into an ultrametric tree using the bladj function of Phylocom [44]. We acknowledge that the resulting chronogram cannot be compared to a dated tree produced by a relaxed clock analysis of original molecular data, but we believe that the benefits of using approximate branch lengths are greater than using topology alone (e.g., all branch lengths equal to one). However, we were careful to use the estimated ages from the study by Magallón and Castillo (2009) [45] as input landmarks for the bladj function, rather than the default age table provided in Phylocom, which is based on an older, now obsolete study of angiosperm divergence times. Specifically, we used as landmarks the mean crown-group ages for orders and the main clades of angiosperms from the constrained analysis reported in Table 1 of Magallón and Castillo (2009) [45].

### Diversity measures

We identified continental scale patterns of species distributions and richness for the woody component (free-standing and climbing taxa) of the rainforest flora by representing species richness as raw data derived from species presences in the 10×10 km grid cells (Fig. 1; and see Fig. 2). Bioregional taxonomic patterns were quantified by sub-setting the continental values into eight regions (Fig. 1). Unless otherwise described, all diversity analyses were undertaken using the software program Biodiverse, version 0.18 [35].

For each 50×50 km grid cell we calculated indices of species richness, phylogenetic endemism (PE) [46], phylogenetic diversity (PD) [47] and phylogenetic structure [48]. These were applied to three species sets: all species, freestanding species only, and climbing species only. For each analysis the tree was pruned to match the respective species set using Biodiverse.

PE is an index that quantifies the degree to which phylogenetic diversity is geographically restricted, and is the phylogenetic analogue of weighted endemism [49]. PE measures the amount of evolutionary time that is restricted to a cell. Here we measure PE relative to species continental distributions only (i.e. as a measure of diversity within Australia; while acknowledging that the range of some taxa extends into Indo-Malesia and that global PE values would potentially be lower for those taxa). Phylogenetic structure was assessed using the Mean Phylogenetic Distance index (MPD). The MPD scores were then standardised into the Net Relatedness Index (NRI) by comparing them to a null community assembly value based on random draws from a larger species pool (both regional and continental pools were assessed). The NRI scores indicate random (neutral), positive (clustering) or negative (evenness) values. The continental pool of species used all species in the respective species set (all, freestanding, climbing), while the regional pools used the same species set but only the species from the region in which the cell occurred. Appendix S1 provides the basis for determining the extent of regions (and see below). Null distributions were calculated for each combination of species richness score for each species set and for each species pool. The null model maintained species richness (by cell) and used random draws from the full (continental) or regional pool of species (for all species combined, and by life form). To generate the null distributions we set 4999 as the default number of iterations, with an early stopping criterion after 500 iterations when the ratio of the minimum to maximum score for the preceding 100 iterations was less than 0.005. The resampling was set to never re-use a sample combination within a pool, so a second early stopping criterion was applied to end the analysis when the maximum possible number of combinations had been reached (see further detail in Appendix S1). The Nearest Related Taxon Index (NTI) was also calculated, but results are not considered here as the focus of this study was to identify and elucidate continental phylogenetic structure signals that reflect the full depth of history in the background phylogenetic tree.

To test the efficacy of our allocation of areas to regions and show the relations between regions (based on floristic similarity-dissimilarity) we used non-metric multidimensional scaling ordination and the Sorensen distance measure (Primer v6) [50]. Outputs from these analyses showed that the merging and expansion of several regions with strong floristic affinities and similar biogeographic history (e.g. Northern Territory and Western Australia; Eungella-Proserpine and Central Queensland; Northern New South Wales and Southeast Queensland) was well founded (Fig. S1 in Appendix S1).

To test the relation of continental gradients of rainfall and temperature to phylogenetic structure (NRI), phylogenetic diversity (PD) and species richness we used a bivariate spatial autoregressive model. Specifically, we used richness, PD and NRI for all taxa combined versus mean annual rainfall and temperature.

## Results and Discussion

### Species richness and taxonomic structures

Rainforest species distribution in Australia (cells with species richness scores from >5 - 464) is plotted with rainfall patterns in Fig 1. The full set of cells (species richness from 1-464) is similar but has much denser cell coverage and extends further inland. Bioregional species richness declined unevenly along the latitudinal gradient, from the northern tropics to southern temperate regions (Table 1; Fig. 2). The seasonally dry tropical monsoon forests of the north and northwest showed considerably lower richness than wet tropical areas in the northeast. However, the more northerly drier vine forests of Central Queensland showed similar richness values to the subtropical moist region further south (Table 1). At continental scale, species richness and phylogenetic diversity (as PD) were highly correlated, *r* = 0.98. The bivariate model showed both richness and PD were significantly positively related to increasing rainfall (*p*<0.0001), but not temperature (Table S3 in Appendix S1).

The signal for latitudinal decline in taxonomic structure and richness in these data is somewhat stronger for vines than trees. In addition, both vines and trees show lower diversity in seasonally drier regions, supporting the idea of higher angiosperm diversity in aseasonal moist tropical and subtropical environments [31], [51]. The taxonomic diversity (Table 1) and phylogenetic dispersion of the climbing habit were relatively broad (Fig. S2 in Appendix S1), but climbers were notably absent from large clades such as Myrtaceae, Lauraceae and Sapotaceae (Appendix S2).

In the tropics the relationship of latitude and rainfall gradients is largely orthogonal, with tropical taxonomic diversity (number of species, genera and families) skewed significantly toward the moist forests of the Wet Tropics (WT) (Table 1). Taxonomic diversity shows a substantial decline in the seasonally drier north and northwest (WA_NT), and a less substantial decline north of the Wet Tropics in seasonally drier areas of Cape York (CY). As latitude increases southwards, taxonomic diversity continues to decline unevenly in response to the interaction of rainfall and latitude gradients. For example, the more highly seasonal and lower rainfall region of north to central Queensland (CQ) has similar levels of taxonomic diversity to the moist central eastern region (BR) at higher latitudes. South of those regions the data show taxonomic diversity declining approximately in line with increasing latitude, from southern New South Wales (SNSW) to Victoria (Vic) and Tasmania (Tas) (Table 1).

### Phylogenetic Endemism

The greatest concentrations of phylogenetic endemism were located in the far north of Queensland (northern section of Cape York including Iron-McIlwraith Range and the Bamaga to Lockerbie areas, and the Wet Tropics), the central eastern subtropics (southeast Queensland and northern New South Wales), and western Tasmania (Fig. 2) [49], [52]. The relative species richness of these regions varied considerably (Table 1). Differences between growth forms were evident as stronger spatial structuring of phylogenetic signals for climbing plants. This was most evident in the concentration of high values for PE in the moist tropics (northeast) and subtropics (central east) (Fig. 2). Interestingly, using free-standing plants alone or in combination with climbing plants identified all of the largest moist rainforest massifs, whereas using climbing plants alone more accurately identified what have often been referred to as ‘core’ areas of moist rainforest refugia in the tropics and subtropics (Fig. 2). In combination, figures 1 and 3 show the moisture gradient (as mean annual rainfall) and its alignment with continental patterns of phylogenetic structure [53]–[55]. In the bivariate model NRI values were significantly negatively related to increasing rainfall (*p*<0.0001) and positively related to increasing temperature (*p*<0.0001) (Table S3 in Appendix S1). That is, they tended toward evenness in relation to rainfall and clustering in relation to temperature.

### Phylogenetic structure relative to continental and regional pools

Comparisons between analyses using regional versus continental pool sizes showed significant differences. The value of using both full continental and regional scale pools to interpret continental patterns of diversity was particularly evident in this case where significant large-scale biogeographic and climate gradients influence species distributions.

Patterns of phylogenetic structure using the continental pool of species, and across the full depth of the phylogeny as measured with net relatedness index (NRI) showed evenness in multiple neighbouring grid cells (species less related than expected by chance) in the widely separated and larger areas of tropical (e.g. Wet Tropics) and sub-tropical rainforest that include moist upland refugia (Fig. 3). In addition the same signal was detected in moist south-eastern forest areas along the Great Dividing Range, and in Tasmania. Phylogenetic signals showed weak to significant clustering in monsoonal (and drier vine) forests in the far north and north-west, and in central Queensland (Fig. 3).

When the same analyses were run using regional species pools, phylogenetic structure (as evenness values for NRI) weakened most noticeably in Tasmania. Central Queensland retained weak to significant clustering, while the southern portion of the central eastern sub-tropical and southeastern warm temperate uplands retained weak to significant evenness. The Wet Tropics region of far north Queensland remained largely unchanged in relation to shifts in pool size with significant evenness in association with coastally aligned upland refugia, and clustering in the nearby western ranges. The Cape York region adjoining the Wet Tropics continued to show significant clustering in the south and a tendency for evenness in multiple grid cells associated with important refugia and Indo-Malesian interactions in the north. Western Australia and the Northern Territory showed weaker and more mixed signals from individual cells at regional pool sizes with phylogenetic structure tending toward neutral overall (Fig. 3).

At continental and regional pool scales climbers showed a relatively strong latitudinal signal for phylogenetic structure (as NRI). Significant evenness was evident only in Wet Tropics and the central eastern sub-tropics, dissipating quickly with increasing latitude to be weakly clustered or neutral in the south (Fig. 3) and represented by few species (Table 1). Patterns for free-standing (tree and shrub) species were very similar to those from analyses using all growth forms combined.

NRI identified seasonally dry regions of the continent where broad-scale phylogenetic structure values were either close to neutral or highly clustered, and moist forest areas with significant non-neutral signals that included both clustering and over-dispersion (evenness). As an example of the latter, the continental scale analyses identified the Dorrigo-New England area in the central eastern subtropics as having high refugial values with both significant evenness (NRI) and high PE [49], a pattern that was less evident using comparisons based on plot-samples at smaller local scales [20]. Interestingly, in the current study, significant evenness (species less related than by chance) was detected both in species-rich areas with high endemism, and species poor areas at higher latitudes with low endemism.

The phylogenetic structure patterns shifted significantly in some regions (e.g. Tasmania) depending on pool size (continental versus regional). Tasmania shares few species with the mainland and has high endemism (at both genus and species levels) despite relatively low diversity. In contrast, the southeast uplands of the Great Dividing Range (in southern New South Wales and Victoria) are part of continuous distributions on continental Australia and have lower endemism, sharing almost all taxa with other areas. In the southeast uplands of the mainland low richness and low relatedness remained consistent across shifts in pool size, while in Tasmania low richness and high regional endemism resulted in a shift from significant evenness at continental pool size to weaker signals at regional pool size (Fig. 3). These patterns are consistent with Tasmania's history of intermittent isolation from the mainland, and are further highlighted by the persistence there of gymnosperm genera that are extinct on the mainland and of significant global palaeobotanical interest (e.g. *Lagarostrobus* and *Phyllocladus* in Podocarpaceae, *Athrotaxis* in Cupressaceae) [53], [54]. Endemic diversity within angiosperm genera such as *Nothofagus* (Nothofagaceae) adds further weight to these patterns [56], [57].

### General synthesis of phylogenetic and assemblage patterns

The overall patterns for phylogenetic structure (NRI) were broadly consistent with previous results from Australian rainforest at (smaller) regional scales using plot-based samples of free-standing species. These showed clustering in historically more disturbed or environmentally filtered areas, and a tendency to increased evenness in stable (core) moist forest refugia such as the Wet Tropics (Fig. 1) [20], [21].

By using full continental sampling, and grid cells rather than samples of plot-based assemblages, the current data provided many additional signals to interpret. The inclusion of all woody rainforest taxa and a comprehensive continental phylogeny allowed us to focus our attention on interpreting the influence of evolutionary and biogeographic history on present-day species distributions and co-occurrence at continental and regional scales.

In relation to the continuous distribution hypothesis, we expected weak phylogenetic structure (NRI close to neutral) and low endemism where continental connectivity was uniformly high because those signals suggest relatively unconstrained dispersal-based assembly and weak habitat filtering. That particular combination of signals was widespread in drier inland, tropical north to northwest seasonally dry, and some species poor southern temperate areas (using regional pools). However, the variation in grid cell values for phylogenetic structure in the monsoon tropics (as an example) suggests a combination of factors influencing distributions and community assembly including connectivity (propagule dispersal) between patches, and environmental filtering.

Recent research suggests large-scale connectivity as an explanation for convergent genetic signals across geographic distance for some taxa described as true rainforest species. For example, genetic studies on the frugivore-dispersed medium to large-seeded *Elaeocarpus grandis* F. Muell. (Elaeocarpaceae) and the wind-dispersed small-seeded *Toona ciliata* M.Roem. (Meliaceae) described low impacts from fragmentation and significant gene flow across landscape scales [23], [28]. Population dynamics for these species demonstrate the potential for relatively quick assemblage-level responses to recent (post-glacial) changes in landscape conditions. To counter that, allied research recognised that other species are constrained to refugia, and could only emerge from their patchy distributions under a somewhat different set of conditions [22]. This leads us to suggest that while interactions between local conditions and species ecologies can constrain species distributions and influence assemblage processes, Australian rainforests still show strong signals of continental scale dynamism and species potential, and are not, as is suggested by the ‘isolated fragments’ hypothesis, universally trapped in refugial isolation.

In relation to the isolated fragments hypothesis, we expected and confirmed strong signals for evenness (over-dispersion) and high endemism values reflecting high rainforest diversity in isolated patches of moist refugia. This included the Wet Tropics of north Queensland, several patches in northern Cape York (e.g. Iron-McIlwraith Ranges), and the central-eastern subtropics. In the case of northern Cape York the signal also included high levels of shared taxa with Indo-Malesia (Fig. S1 in Appendix S1). The tendency to evenness (at continental and regional pool sizes) and high values for PE in western Tasmania also identified isolated refugia. In contrast, areas inland from and adjacent to the tropical and subtropical moist forest refugia showed stronger clustering and lower endemism values despite relatively high diversity, suggesting strong environmental filtering of more similar and related taxa into shared habitats. A similar pattern was evident for seasonally drier areas of central Queensland where we assumed environmental filtering was strong, and diversity remained relatively high.

In relation to the continuous distribution hypothesis, we detected significant evenness (over-dispersion) and low endemism values in southeast upland areas (SNSW and Vic). Despite low rainforest species richness, and dominance by moist sclerophyll vegetation types, this area showed significant phylogenetic structure (as evenness for NRI). However, it was not identified by PE as an area where substantial components of phylogenetic diversity were restricted. Instead the results show that a suite of rainforest woody species drawn from across the phylogeny were widespread, and despite low richness overall the area retained important continental rainforest diversity. Perhaps most importantly these patterns demonstrate the potential for rapid response by rainforest species to changing conditions, including expansion and contraction, from a local but ‘connected’ species pool.

At continental scales, we expected that clustering and high endemism could co-occur where there were high levels of speciation in taxa filtered into shared habitats. However, this was not a strong signal in the data suggesting that in situ diversification has not been a major factor shaping the current rainforest flora. For example, while there were some signals of clustering in the monsoon tropics at continental pool size (Fig. 3), the associated signals for endemism in adjacent cells in the same areas were weaker (Fig. 2). In Australia, continental signals of lability and diversification have most often been predicted for recently (ca. 20 Ma to present) expanded vegetation types such as fire and dry-adapted sclerophyll communities [58], rather than rainforest. In fact, the rainforest biome has been suggested as inherently conservative [19].

In this study we expected the signal of lability and diversification to be present in more recently colonised lowland areas of the monsoon (seasonally dry) and moist tropics in northern Australia, and the drier vine forests of Central Queensland, where a more recent history of invasion by floral elements from Indo-Malesia has previously been postulated [13], [29]. The proposed relationship between increasing Indo-Malesian floristic elements and decreasing moisture in drier (seasonal) tropical forests was confirmed (in part) in this study.

Our results showed that southern Cape York and Central Queensland both had significant phylogenetic clustering (NRI), potentially indicating some recent arrival and speciation, but perhaps more likely suggesting the influence of stronger environmental filtering (e.g. [59]). Only Cape York had consistently high values for PE, but this was mostly in the north of the region, where NRI values showed more evenness and both moist forest refugia and Indo-Malesian interactions were present. The monsoon tropics of Western Australia and Northern Territory showed mixed signals with generally lower values for PE; and NRI tended to clustered values using the continental pool and more neutral values using the regional pool. Like Cape York, the region has high levels of taxa shared with Indo-Malesia (Table 1). In addition, the signal of increased evenness from the upland-lowland wet tropics confirmed the potential for more recent Indo-Malesian origins (or interactions) for some taxa (e.g. fast-growing disturbance-related genera such as *Macaranga* in Euphorbiaceae on the tropical lowlands). However, taken together the findings suggest a complex biogeographic and evolutionary history for these areas involving a mix of conservative Gondwanan and more recent Indo-Malesian influences and interactions.

Previous (finer-scale plot-based) studies showed significant species turnover along the altitudinal gradient in the Wet Tropics but little shift in community phylogenetic values [20]. Strong genus-level connections between mature-phase (shade-tolerant) taxa from upland and lowland areas were subsequently confirmed. This suggests that (in the current study) the signal of speciation along the gradient from moist forest refugia in the uplands [10], [60] to the tropical lowlands was partly obscured in the phylogenetic signal by close relatedness [20]. In addition, because grid cells can include both upland and lowland areas, the larger assemblage samples (grid cells) used here may have concealed some finer-scale phylogenetic differentiation along the altitudinal gradient. Finer-scale sampling and genetic research into Australian rainforest taxa has provided evidence of isolation and drift relative to environmental gradients [24], [61] and will continue to yield important new insights into regional relationships through time.

We found only partial support for the ‘isolated fragments’ and ‘continuous distribution’ hypotheses, but a strong impetus to merge them and define a new hypothesis for Australian rainforest distributions. This more parsimonious ‘continental connectivity’ hypothesis describes the Australian rainforest as a structured continuum of rainforest species and assemblages distributed along two often orthogonal continental axes of variation (reflecting wet to seasonally dry and tropical to temperate habitats) with an archipelago of higher and lower diversity and different sized patches embedded in it.

We argue that the rainforests, and many rainforest species, retain the ability to respond relatively rapidly to changing condition across the landscape, as evidenced by this and previous phylogenetic, evolutionary, palaeobotanical and ecological research [14], [20], [21]. However, we acknowledge that this ability has been eroded by more recent clearing and forest modification. The challenge is to safeguard what remains of that potential by protecting continental dynamics and the continuous landscape matrix in which the islands of rainforest diversity are embedded [62].

From a regional perspective, the results have laid the full matrix of extant continental rainforest woody species distributions over the shapes formed by evolutionary and biogeographic history. In so doing, they have described (for one continent at least) a temporal geographic landscape that will help set research agendas and conservation targets based on addressing how rainforest evolutionary history and current day phylogenetic and functional (trait) diversity are spread and interact across regional, continental and global scales.

The potential to include whole of continent patterns of phylogenetic structure into global interpretations of rainforest origins, evolution, and biogeography is very recent. Although considerable challenges remain in relation to data compilation, taxonomic resolution, and phylogenetic tree building, the convergence of factors that enabled this study represent advances in systematics and phylogeny, community phylogenetics, spatial ecology, and bioinformatics that are rapidly expanding the horizon of researchers to expand to continental and global scales.

## Supporting Information

Appendix S1 **Floristic analysis (Sorensen, NMDS) and regional allocation (Fig. S1)**; 15 most important families and genera in Australian rainforest by growth form (Tables S1 and S2); summary results of bivariate autoregressive spatial models of environmental variables (mean annual rainfall and temperature by grid cell) to Species Richness, PD and NRI values (Table S3); background phylogenetic tree by growth form (free-standing and climbing species) (Fig. S2).
(DOC)

Appendix S2 **Ultrametric phylogenetic tree used in the analyses of phylogenetic endemism, diversity, and structure.** Growth form mapped using parsimony in Mesquite 2.75. Time scale in million years.
(PDF)
