# Supplementary material for: Landscape Patterns in Rainforest Phylogenetic Signal: Isolated Islands of Refugia or Structured Continental Distributions?
Source: PLoS One. 2013 Dec 2;8(12):e80685. doi: 10.1371/journal.pone.0080685 (PMC3846590; doi:10.1371/journal.pone.0080685)
Supplement: Appendix S1 — Floristic analysis (Sorensen, NMDS) and regional allocation (Fig. S1); 15 most important families and genera in Australian rainforest by growth form (Tables S1 and S2); summary results of bivariate autoregressive spatial models of environmental variables (mean annual rainfall and temperature by grid cell) to Species Richness, PD and NRI values (Table S3); background phylogenetic tree by growth form (free-standing and climbing species) (Fig. S2). (DOC) [file pone.0080685.s001.doc]

**APPENDIX 1 - SUPPORTING MATERIALS**

Landscape patterns in rainforest phylogenetic signal: isolated islands of refugia or structured continental distributions?

Kooyman, R.M., Rossetto M., Sauquet, H. and Laffan, S.W.

**Floristics and regions**

Multivariate methods (clustering analysis and non-metric multidimensional scaling ordination NMDS) were used to assess the relationship of geographical regions. In addition, a derived group representing a composite of species known to occur on the Australian mainland (and in-shore islands) and in Indo-Malesia (including Papua New Guinea) was generated and included in the analysis to determine floristic affinities. Geographical regions were determined by reference to 1) the eco-floristic regions and vegetation provinces identified by Webb and Tracey (1979, 1981), 2) geographical boundaries representative of previously described rainforest distributions (e.g. Webb & Tracey, 1981; Adam, 1992), and 3) some minor separation based on political boundaries (e.g. southern New South Wales and Victoria). The a priori geographic regions include the north of Western Australia (Kimberley Region of WA), Northern Territory (NT), Cape York (CY), Wet Tropics (WT), Eungella-Proserpine, Central Queensland (CQ) to south-east Queensland (SEQ), northern New South Wales (NNSW), Central New South Wales (CNSW), southern New South Wales (SNSW), Victoria (Vic) and Tasmania (Tas).

The following NMDS ordination based on Australian woody rainforest taxa shows floristic relations between bio-regions similar to those described by Webb and Tracey (1979; 1981a,b; Webb *et al*., 1984). The (NMDS) ordination shows the strength of relations between major rainforest areas as groupings based on a triangular resemblance matrix (generated using Sorensen similarity) derived from floristic data (as a rectangular matrix of site by species) for all free-standing and climbing woody rainforest species distributions. To test if differences between life forms were significant, free-standing and climbing species were analysed independently. Results (not presented) showed that patterns were similar to the merged analysis shown below (Fig. S1).

The tropical region includes the west Australian Kimberley Region (WA), Northern Territory (NT), Cape York (CY), and Wet Tropics in Far North Queensland (FNQ). Strong grouping between WA and NT supported them being treated as a single region. Species that are shared with Indo-Malesia (including PNG) group most strongly with CY. The sub-tropical grouping includes the Eungella Range and Proserpine area (Eung-Pros), parts of central Queensland (CQ) to south-east Queensland (CQ-SEQ) and northern New South Wales (NNSW). However, there is some partitioning of the group relative to latitude (north-central versus southern). The sub-tropical influence extends southward toward central (C_NSW) and southern NSW (SthNSW) where it interacts with the more temperate rainforest grouping that includes southern NSW and Victoria (Vic). Tasmania (Tas) remains as a cool-temperate outlier to the other groupings.


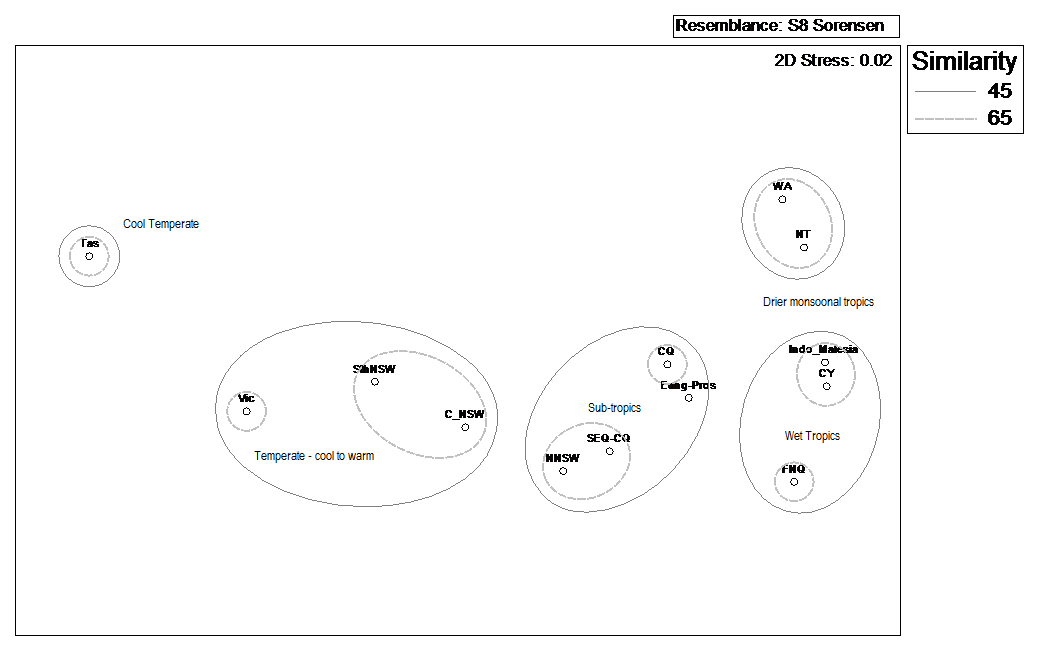


Fig. S1 NMDS ordination showing relationship of Australian rainforest regions based on the distribution of all known free-standing and climbing woody rainforest species. Ellipses represent 45% and 65% similarity based on the underlying triangular resemblance matrix. WA - West Australian Kimberley Region; NT - Northern Territory; CY - Cape York; WT - Wet Tropics in Far North Queensland (FNQ); Indo-Malesia (includes PNG); Eung-Pros - Eungella Range and Proserpine area; CQ - Central Queensland; CQ-SEQ - Central Queensland to south-east Queensland; NNSW - Northern New South Wales; C_NSW - central New South Wales; SthNSW - southern NSW; Vic – Victoria; and Tas - Tasmania.

**Null model for NRI discussion**

We set 4999 as the default number of iterations, with an early stopping criterion being applied after 500 iterations when the ratio of the minimum to maximum score for the preceding 100 iterations was less than 0.005. The resampling was set to never re-use a sample combination within a pool, so a second early stopping criterion was applied to end the analysis when the maximum possible number of combinations had been reached.

The application of this approach resulted in very few samples converging in fewer than 1300 iterations. This has possible implications for analyses where the iterations parameter is fixed at 999. Of those samples which converged in fewer than 999 iterations, all except the Tasmanian vine species pool needed greater than 900 for richness scores of 2, 3 or 4 (Cape York, WA/NT and Victoria vine species pools and the Tasmanian freestanding species pool).The Tasmanian vine species had a pool size of only 6 species and richness scores of only 2, 3 and 4 and therefore the maximum number of combinations were used in the analyses.

Table S1 Fifteen most diverse angiosperm families of woody free-standing (trees and shrubs) and climbing (vines) plants in Australian rainforest defined by number (n) of genera and species.

| TREES |  |  |  | VINES |  |  |
| --- | --- | --- | --- | --- | --- | --- |
| family | genera(n) | species(n) |  | family | genera(n) | species(n) |
| Fabaceae | 32 | 80 |  | Fabaceae | 20 | 50 |
| Myrtaceae | 31 | 187 |  | Apocynaceae | 17 | 98 |
| Proteaceae | 31 | 78 |  | Menispermaceae | 13 | 26 |
| Rubiaceae | 28 | 103 |  | Convolvulaceae | 10 | 26 |
| Sapindaceae | 27 | 138 |  | Cucurbitaceae | 10 | 17 |
| Euphorbiaceae | 26 | 84 |  | Rubiaceae | 6 | 18 |
| Malvaceae | 26 | 88 |  | Annonaceae | 5 | 15 |
| Rutaceae | 26 | 100 |  | Rhamnaceae | 5 | 7 |
| Cunoniaceae | 12 | 29 |  | Vitaceae | 5 | 28 |
| Celastraceae | 11 | 26 |  | Euphorbiaceae | 4 | 6 |
| Ericaceae | 11 | 28 |  | Lamiaceae | 4 | 5 |
| Meliaceae | 11 | 43 |  | Araliaceae | 3 | 3 |
| Phyllanthaceae | 11 | 69 |  | Bignoniaceae | 3 | 8 |
| Annonaceae | 10 | 29 |  | Celastraceae | 3 | 6 |
| Apocynaceae | 10 | 34 |  | Moraceae | 3 | 3 |

Table S2 Fifteen most diverse angiosperm genera in Australian rainforests defined by number (n) of species in each genus of free-standing (trees and shrubs) and climbing (vines) plants.

| TREES |  |  |  | | VINES | |  | |  | |  |
| --- | --- | --- | --- | --- | --- | --- | --- | --- | --- | --- | --- |
| genus | in family | species(n) | |  | | genus | | in family | | species(n) | |
| *Syzygium* | Myrtaceae | 74 |  | | *Parsonsia* | | Apocynaceae | | 28 | |  |
| *Cryptocarya* | Lauraceae | 51 |  | | *Marsdenia* | | Apocynaceae | | 24 | |  |
| *Ficus* | Moraceae | 42 |  | | *Ipomoea* | | Convolvulaceae | | 14 | |  |
| *Endiandra* | Lauraceae | 40 |  | | *Cissus* | | Vitaceae | | 12 | |  |
| *Elaeocarpus* | Elaeocarpaceae | 29 |  | | *Caesalpinia* | | Fabaceae | | 10 | |  |
| *Diospyros* | Ebenaceae | 22 |  | | *Tylophora* | | Apocynaceae | | 9 | |  |
| *Croton* | Euphorbiaceae | 21 |  | | *Cayratia* | | Vitaceae | | 8 | |  |
| *Acronychia* | Rutaceae | 20 |  | | *Hoya* | | Apocynaceae | | 8 | |  |
| *Gossia* | Myrtaceae | 19 |  | | *Jasminum* | | Oleaceae | | 8 | |  |
| *Myrsine* | Primulaceae | 19 |  | | *Morinda* | | Rubiaceae | | 8 | |  |
| *Symplocos* | Symplocaceae | 19 |  | | *Pararistolochia* | | Aristolochiaceae | | 7 | |  |
| *Rhodamnia* | Myrtaceae | 18 |  | | *Piper* | | Piperaceae | | 7 | |  |
| *Brachychiton* | Malvaceae | 16 |  | | *Rubus* | | Rosaceae | | 7 | |  |
| *Cupaniopsis* | Sapindaceae | 16 |  | | *Smilax* | | Smilacaceae | | 7 | |  |
| *Dysoxylum* | Meliaceae | 16 |  | | *Clematis* | | Ranunculaceae | | 6 | |  |

Table S3 Summary results of bivariate autoregresive spatial model for mean annual rainfall and temperature (by grid cell) and richness – Species Richness, PD – Phylogenetic Diversity, and NRI – Net Relatedness Index; *b* – slope of relationship, *p* – significance value.

|  | *b* (rain) | *p* (rain) | *b* (temp) | *p* (temp) |
| --- | --- | --- | --- | --- |
| richness | 0.142 | 0.000 | -1.202 | 0.667 |
| PD | 6.069 | 0.000 | -79.710 | 0.506 |
| NRI | -0.001 | 0.000 | 0.180 | 0.000 |


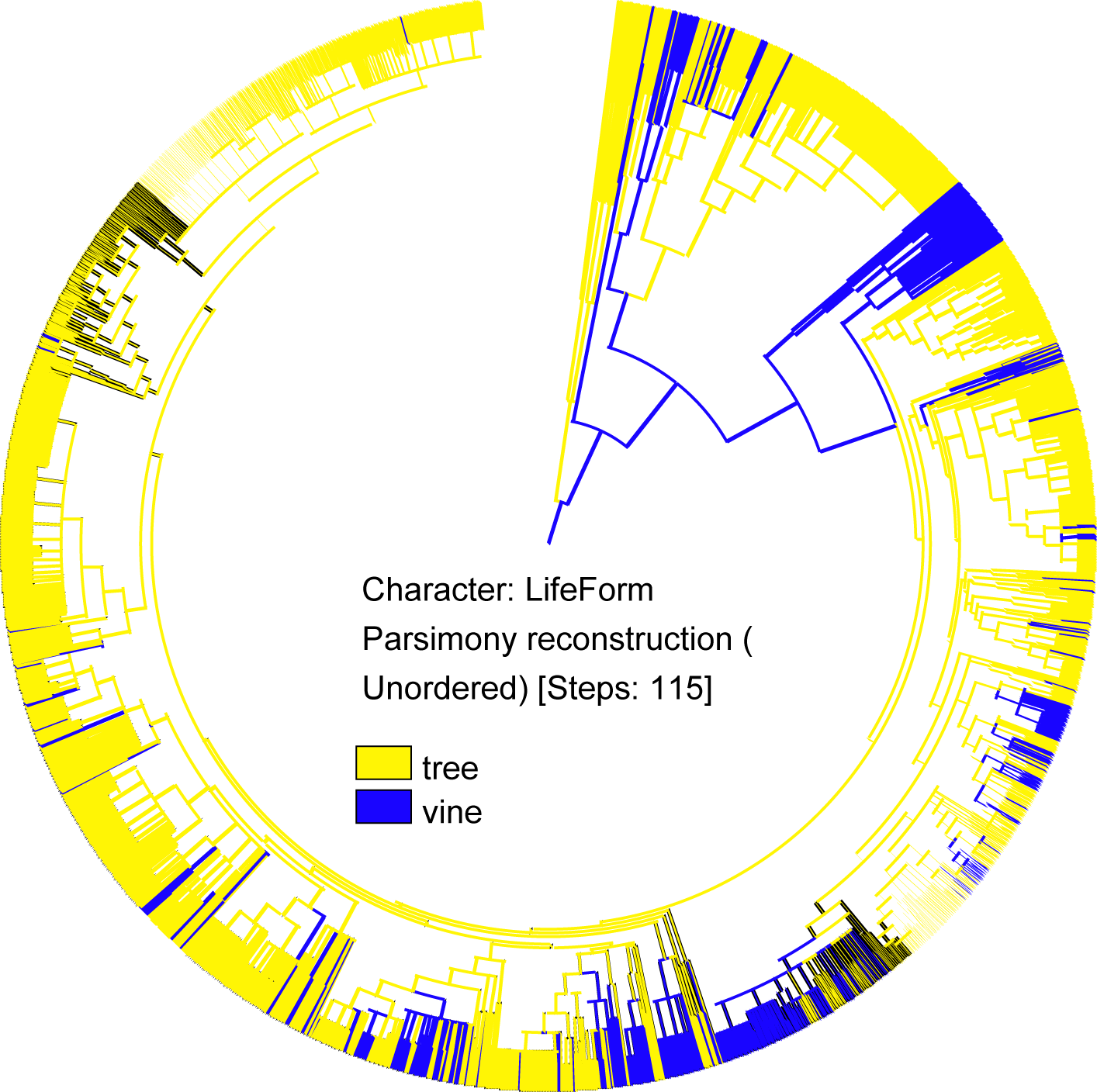


Fig. S2 Background phylogenetic tree showing the dispersion of growth habit (free-standing - tree; and climbing - vine) across the phylogeny. This trait was optimized using parsimony in Mesquite 2.75 (see Appendix File S2 for a detailed version of the same tree).
