# Supplementary figures and images for: Landscape Patterns in Rainforest Phylogenetic Signal: Isolated Islands of Refugia or Structured Continental Distributions?
Source: PLoS One. 2013 Dec 2;8(12):e80685. doi: 10.1371/journal.pone.0080685 (PMC3846590; doi:10.1371/journal.pone.0080685)

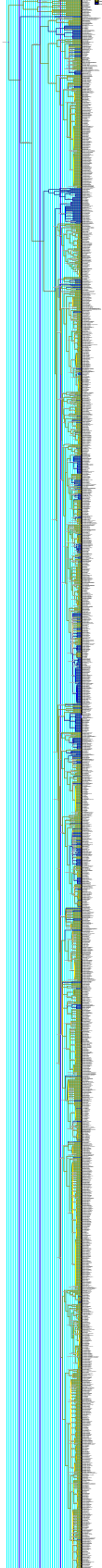

Supplement: Appendix S2 — Ultrametric phylogenetic tree used in the analyses of phylogenetic endemism, diversity, and structure. Growth form mapped using parsimony in Mesquite 2.75. Time scale in million years. (PDF) [file pone.0080685.s002.pdf]
